# Assessment of oral cytological changes associated with exposure to chemotherapy and/or radiotherapy

**DOI:** 10.4103/1742-6413.51332

**Published:** 2009-05-16

**Authors:** Hussain G Ahmed, Dalia AI Elemirri

**Affiliations:** Department of Histopathology and Cytology, Faculty of Medical laboratory Sciences, University of Khartoum, Khartoum, Sudan

**Keywords:** Atypia, chemotherapy, cytology, oral, mucosa, radiotherapy

## Abstract

**Background:**

Death from cancer is high in Sudan, with low survival rates, as most of the patients present with advanced disease. Most patients receive high and repeated doses of radiotherapy or chemotherapy. The aim of this study was to investigate the feasibility of using cytological evaluation to detect oral epithelial atypia amongst these patients. As a part of the continuous development in cancer therapy, this case control study was conducted in Khartoum, Sudan.

**Methods:**

Papanicolaou stained oral mucosal cells were obtained from 100 cancer patients receiving radiotherapy and/or chemotherapy (ascertained as cases), 50 cancer patients not exposed to either therapy (control 1), and 50 apparently healthy individuals (control 2).

**Statistical analysis:**

The data was analyzed by using a computer SPSS program, to obtain the Chi-square test.

**Results:**

Without prior knowledge of the subjects' group, oral epithelial atypia was detected in 7% of the cases. Inconclusive features of cytological atypia were observed in 13% of the cases. Atypia was not observed in both the control groups. Inflammatory infiltrate and viral cytopathic effects were identified in 32% and 8% of the cases respectively.

**Conclusion:**

Cytological atypia, viral infections, and inflammatory infiltrates were detected after exposure to radiotherapy and/or chemotherapy.

## INTRODUCTION

Early detection and follow-up of oral precancerous lesions is important for predicting their potential for malignancy.[1] The primary risk factors for the development of these lesions are comparable worldwide.[1,2] Many reports have described various cytoplasmic and nuclear changes after radiation therapy. These changes include cellular enlargement, vacuolization, cytoplasmic granulation, nuclear enlargement, pyknosis, karyorrhexis, karyolysis, multinucleation, micronucleation, nuclear budding, and binucleation.[3–5] One of the most severe side effects of radiation is oral mucositis.[6] Chemotherapy is also known to cause effects on the oral mucosa, such as a decreased renewal rate of the basal layer of epithelial cells that line the oral cavity.[7] The oral mucosal changes may differ, subject to many factors such as dose concentration, cycles, and type of treatment.

The exfoliative cytology is conventionally used for screening and diagnosis of oral mucosal lesions,[8,9] it may also be applied and preferred over clinical assesment to monitor therapy related changes.

## MATERIALS AND METHODS

In this study, the effects of radiotherapy and/or chemotherapy were assessed on oral mucosa by cytology, during the period from January 2007 to August 2007. The study was conducted at the Radio Isotope Centre, Khartoum. The study subjects were cancer patients and non-cancer volunteers living in the city of Khartoum, Sudan. Two hundred individuals were selected by a random method, among whom 100 were cancer patients on radiotherapy and/or chemotherapy (assigned as case group), 50 were cancer patients not receiving radiotherapy or chemotherapy (assigned as control 1), and 50 were apparently healthy subjects (ascertained as control 2). Of the 100 cases, 56 (56%), 7 (7%), and 37 (37%) were patients receiving chemotherapy, radiotherapy, and both therapies respectively. 100 cancer patients included head and neck cancers (27), breast cancer (30), hematolymphoid malignancies (13), cervical cancer (10), prostatic cancer (10), and the remaining 10 patients had tumors with unknown primary.

Control group 1 included cases with head and neck cancers (1), hematolymphoid malignancies (10), breast cancer (12), cervical cancer (13), prostate cancer (7), and other cancers. Regardless of the gender, the study subjects ranged from10 to 80 years of age. Oral cancer patients, with bad oral hygiene, tobacco users and alcohol dependance were excluded from the study.

### Selection criteria

All patients (case group and control 1) who fulfilled the selection criteria were given serial numbers. The participants were selected by the addition of a constant number that gave equal chance for each one of the study population to be selected. This was achieved by the use of a random method, in which the sample interval is determined by dividing the sample size on the study group. The sample interval in this study was found to be 15, then the number of the first patient to be included in the sample was chosen randomly by blind picking of one of the 15 pieces of papers numbered 1 to 15. The number picked was 8; thereafter, every 15^th^ number was added, starting from patient number 8, to be selected as the study subjects. Consequently, the numbers of the selected patients were: 8, 23, 38, and so on, up to the selection of 100 cancer patients on cancer therapy or 50 cancer patients not exposed to therapy. Control 2 represented all apparently healthy individuals who visited the oncology centre as patient companion who volunteered to participate.

#### Ethical consent:

Each participant was asked to sign a written ethical consent during the interview, before the specimen was taken.The informed ethical consent form was designed and passed by the ethical committee of the faculty research board.

#### Sample size:

The sample size was calculated using software known as the survey system, available at http://www.surveystem.com.sscalc.htm. The system inertly relies on the equation: *n* = z^2^pq/d^2^ (*n* = sample size; z = the standard normal deviate, usually set at 1.96, which corresponds to the level of the 95% confidence level; p = the proportion to the target population i.e. percentage of the studied group, which is 0.11 in this study; q = 1.0 – p). As the case group and control 1 subjects were selected from the cancer patients who presented during the period from January 2007 to August 2007, the sample size was calculated from the total number of the patients treated at the cancer center, and was found to represent about 11% of the total surviving cancer patients in Sudan. On the other hand, the sample size for control 2 was set as 50, without referring to a specific equation for calculation, and they represented the apparently healthy individuals.

#### Sample collection:

Cytological smears of exfoliative cells were collected from buccal mucosa (covering both cheeks) by brush and the obtained materials were directly smeared on clean glass slides and immediately fixed in 95% ethyl alcohol, while they were wet, and sent to the cytopathology laboratory for further processing. A specimen was taken from a case after exposure to one cycle of radiotherapy and/or chemotherapy.

#### Sample processing:

The smears were stained using the Papanicolaou staining method. Ethyl alcohol fixed smears were hydrated in descending concentrations of 95% alcohol through 70% alcohol to distilled water, for two minutes in each stage. Then the smears were treated with Harris' hematoxylin for five minutes to stain the nuclei, rinsed in distilled water and differentiated in 0.5% aqueous Hydrochloric Acid for a few seconds, to remove the excess stain. They were then immediately rinsed in distilled water, to stop the action of discoloration. Then the smears were blued in alkaline water for a few seconds and dehydrated in ascending alcoholic concentrations from 70%, through two changes of 95% alcohol for two minutes for each change. The smears were next treated with Eosin Azure 50 for four minutes. For cytoplasmic staining, they were treated with Papanicolaou Orange G6 for two minutes, rinsed in 95% alcohol and then dehydrated in absolute alcohol. The smears were then cleared in Xylene and mounted in DPX (Distrene Polystyrene Xylene) mount. All the reagents used were from Thermo Electron Corporation, UK.

#### Assessment of the results:

**Figure 1:**
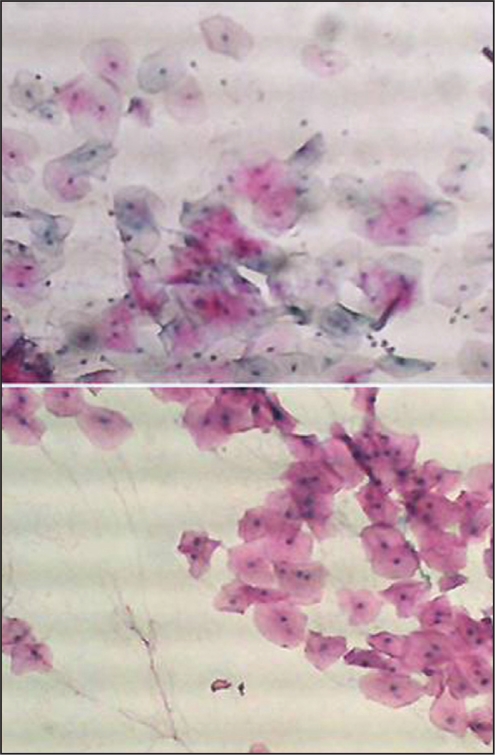
Buccal smear from a patient with head and neck cancer non-exposed to chemotherapy or Radiotherapy. Normal epithelial cells. Pap. × 10

To increase the reliability and reproducibility, strict quality control measures were applied. We included 10 smears from patients with histopathologically diagnosed oral cancer to serve as positive control. In assessing the quality of staining, the smears were examined under low (10X) power using a light microscope. All included smears showed satisfactory staining quality with blue nuclei, pink/orange cytoplasm of the keratinized squamous cells and blue/green staining of the cytoplasm of the non-keratinized squamous epithelial cells, as shown in Figure 1. To avoid the assessment bias, cytological smears were labeled in such a way that the examiner was blinded to the groups (case group, control 1, or control 2) of each subject.

Atypia was assessed cytologically by using the criteria described elsewhere.[10] The presence of two or more of the following features were consistent with atypia: nuclear enlargement associated with increase nuclear cytoplasmic ratio, hyperchromatism, chromatin clumping with moderately prominent nucleoli, irregular nuclear membranes and bi or multinucleation, scant cytoplasm, and variation in size and/or shape of the cells and nuclei. The percentages used below were calculated by counting normal and atypical cells in five microscopic fields using 40X objective. The mean of the count was then calculated for both types of cells. The two means were added together, and the total mean value of cells counted per field was generated.

If the atypical cells were 3-10%, this was considered slight and given a score value as follows: for 3-6% atypical cells, the score assigned was (+) and for 7-10% the score was (++). More than 10 atypical cells was consistent with marked atypia. The marked changes were scored as follows: 10-15% was (+) for mild cytological atypia; 16-20%, was (++) for moderate cytological atypia, and 21%, was (+++).

### Data analysis

Statistical Package for Social Sciences (version 10) was used for analysis and to perform Pearson Chi-square test for statistical significance (*P* value). The 95% confidence level and confidence intervals were used.

## RESULTS

The description of the cases and the controls by demographic characteristics is shown in Table 1. The age distribution was relatively similar among the cases and the controls. The mean age of the study population was 43 years, with a range of 10 to 80 years. The study group consisted largely of females (139 females and 61 males). The vast majority of the cases belonged to the 40-49 year age group and constituted 29% of the cases. Whereas, a majority of the patients from control 2 group were in the 20-29 years age range, and represented 60% of the patients, as shown in Table 1.

**Table 1:** Description of the cases and controls by demographic characteristics

*Age group*	*Cases†*	*Control 1‡*	*Control 2§*	*Females*	*Males*
10-19	3	10	0	9	4
20-29	8	30	1	28	11
30-39	13	6	12	15	8
40-49	29	3	14	31	13
50-59	22	1	14	26	11
60-69	15	0	5	16	14
70+	10	0	0	14	0
Total	100	50	50	139	61

† Cancer patients on radiotherapy and/or chemotherapy;

‡ Cancer patients not on radiotherapy or chemotherapy;

§ Apparently healthy subjects

Epithelial atypia was detected in seven patients, with two patients having severe atypia, two with moderate atypia and three with mildly atypical changes. Notably, there were thirteen cases with borderline atypia. Consequently, the risk of epithelial atypia associated with radiotherapy or/and chemotherapy was found to be statistically significant, by Chi-square test (*P*<0.0001). However, three (42.8%) cases of atypia were discovered among the radiotherapy group, two (28.6%) in the chemotherapy group and two (28.6%) in the group receiving both the therapies. Notably, no evidence of any cytological atypia was detected among both the control groups.

**Figure 2:**
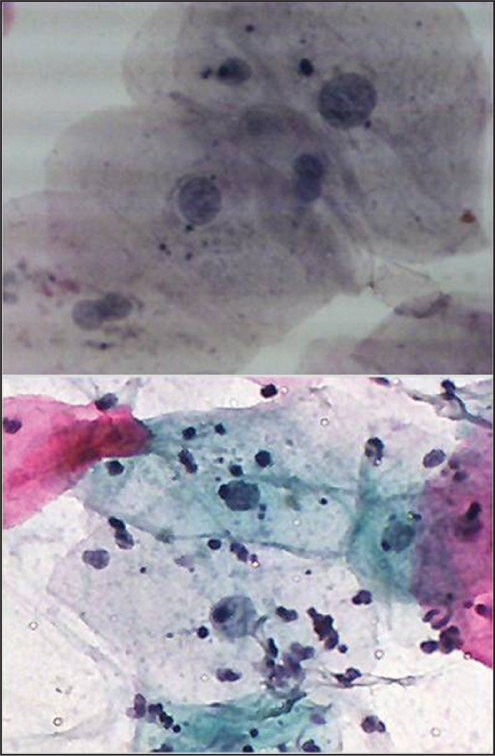
Buccal smear from a patient receiving Radiotherapy with head and neck cancer. Moderate cytological atypia. Increased keratinization with increased N/C ratio and background containing inflammatory cells. Pap. Stain 40×

Regarding the number of radiotherapy cycles (doses) and the degree of cytological atypia, for cycles group (21-5), three cases were detected as having slight cytological changes, one case with mild atypia, and one showed moderate atypia. For cycles group (16-20), three cases showed slight cytological changes. For cycles group (11-15), four cases had mild atypia, while one showed moderate atypia. For cycles (1-5), one case showed mild atypia as shown in Figure 2.

With respect to chemotherapy cycles, for cycles group (16-20), one case showed slight cytological changes. For cycles group (6-10), one case was found with mild atypia, one with moderate atypia, and seven with slight cytological changes. For cycles group (1-5), one case showed severe atypia, one showed mild atypia, and four showed slight cytological changes.

Furthermore, an acute inflammatory cells infiltrate was detected in 17 (17%) of the cases and three (6%) of the cases in the control 2 group. Chronic inflammatory cells were present in 15 of the cases. These results showed statistical differences, by Chi-square test (*P*<0.0001).

With reference to the relation between therapy and inflammatory infiltrates, inflammatory infiltrates were detected in 32 (32%) of the cases, as compared to only 3 (3%) in the control groups, as shown in Table 2. Acute inflammation was found in 12 (21.4%) patients who received chemotherapy, 2 (28.6%) patients who received radiotherapy, and 3 (8%) patients who received both the therapies. Chronic inflammation was found in 4 (7%)patients who received chemotherapy and in 11 (29.7%) patients who received both the therapies as shown in table 2.

**Table 2:** Distribution of cases by therapy and inflammatory infiltrate

*Inflammation*	*Chemotherapy*		*Radiotherapy*		*Both*	
			
	*No.*	*%*	*No.*	*%*	*No.*	*%*
None	40	71.4	5	71.4	23	62.3
Acute	12	21.4	2	28.4	3	8
Chronic	4	7	0	0	11	29.7
Total	56	100	7	100	37	100

Cytological features of viral infection were detected among 8 (4%) cases, of whom 6 (3%) cases were from the chemo-radiotherapy group, and 2 (1%) were within the chemotherapy group. No evidence of viral infection was detected in either control groups. The difference was statistically significant, as seen by the Chi-square test (*P* = 0.004).

## DISCUSSION

To assess the effects of chemotherapy and/or radiotherapy in countries with limited resources such as Sudan, exfoliative cytology is used as a simple and cost effective method. This method might be ideal for screening the risk population. In this study, cytological atypia was quantified in smears obtained from patients exposed to cancer therapies and compared to the non-exposed individuals. This is the first report from the Sudan to use oral exfoliative cytology for the assessment of cytological atypia in smears obtained from patients exposed to cancer therapy.

**Figure 3:**
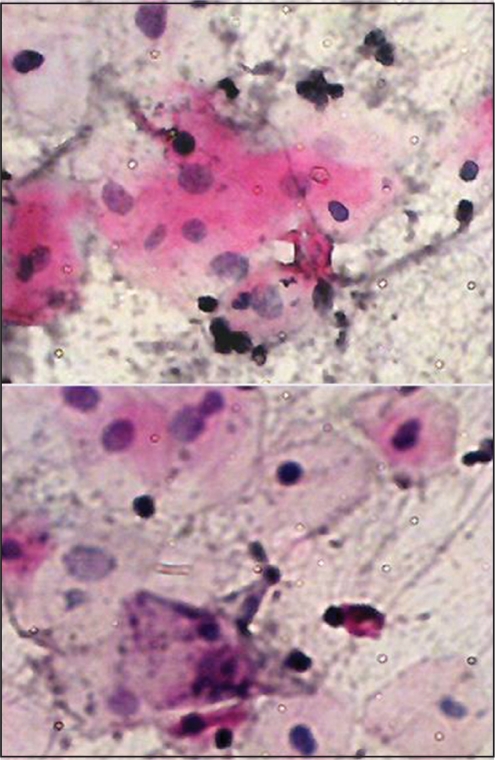
Buccal smear from a patient receiving chemotherapy with carcinoma of hypopharynx. Moderate cytological atypia. Increased keratinization with increased N/C ratio, Hyperchromasia and background containing inflammatory cells. Pap stain ×40

We found cytological atypia in smears obtained from 20 patients of those exposed to therapies, while no atypia was detected in non-exposed subjects. Of these, 7 patients had evidence of cytologic atypia, while 13 had only borderline changes. These findings suggest that chemotherapy and/or radiotherapy may be associated with oral cytological atypia, due to their ability to destroy rapidly proliferating cells. Many cytological features have been reported in the literature.[4,5,11,12] Our findings suggest that these cellular changes may be linked to the factors such as cycle and dose of therapeutic agents [Figure 3].

When we examined the association between the site of cancer and cytological atypia, 75% of the patients showing cytological atypia with slight cytological changes had head and neck cancers. Factors such as type of cancer, type of the chemotherapy, duration, and dose may require further evaluation.

Oral exfoliative cytology has been used for the assessment of epithelial atypia[10,13] and screening for early diagnosis of premalignant and malignant oral mucosal lesions.[14–17] It is known that the exfoliated cells are from the superficial layer, while most of the atypical cells are located in the deeper layers. The degree of cytological atypia varied in many studies based on qualitative evaluation.[18,19] To increase the reliability and to reduce the interference by cytology processing, we used a more specific scoring system,[10,13,20] including consideration for karyolysis, karyorrhexis, pyknosis, and cytolysis. These features were not considered as atypia in this study, but they were noted[3] as a part of the atypical changes in quantifying the epithelial atypia induced by radiotherapy.

When examining the association between the number of radiotherapy cycles and the degree of epithelial atypia, it was found that high proportions of severe epithelial atypia was observed among those exposed to a higher number of radiotherapy cycles. When grouping the number of cycles into two main categories – 20 cycles or more, and less than 20 cycles, cytological atypia appeared to worsen with the increasing exposure, although this was not statistically significant. With chemotherapy, severe epithelial atypia was observed among those exposed to a fewer treatment cycles. This comparison, however, is based on a few patients with higher number of chemotherapy treatment cycles.

We found increased inflammatory infiltrates in post-therapeutic smears, as compared to non-treated patients [Table 2]. This suggests the role of chemotherapy and radiotherapy in inducing inflammatory changes in the buccal mucosa. Chronic inflammatory infiltrates were increased amongst those who received both therapies, and were less prominent in those who received chemotherapy alone. This increase in chronic inflammatory infiltrates may be due to the fact that the patients were immuno-compromised, and were susceptible to different infections.

A number of reports have shown that medical treatments that have cytotoxic effects on lymphocytes affecting cytokines production increase the risk of viral infections. The reported incidence of herpes simplex virus type I infection during radiotherapy is 29.1%.[21,22] Susceptibility to infections of the oral mucosa to a number of other viruses, including Cytomegalovirus, Varicella Zoster and Epstein virus, have also been reported.[23,24]

## CONCLUSION

Radiotherapy and/or chemotherapy induce variable degrees of atypical cytological changes. Local infections especially viral infections are the most common. Oral exfoliative cytology can detect cytological atypia that frequently appear in premalignant oral lesions. In our experience, oral exfoliative cytology is an approrriate method to follow atypia in patients undergoing radiation chemotherapy for oro-facial lesions especially in developing countries with limited resources.

## COMPETING INTEREST STATEMENT BY ALL AUTHORS

No competing interest to declare by any of the authors

## AUTHORSHIP STATEMENT BY ALL AUTHORS

All authors of this article declare that we qualify for authorship as defined by ICMJE http://www.icmje.org/#author.

Each author has participated sufficiently it the work and take public responsibility for appropriate portions of the content of this article.

Each atuhor acknowledges that this final version was read and approved.

## References

[1] Johnson NW, Ranasinghe AW, Warnakulasuriya KA (1993). Potentially malignant lesions and conditions of the mouth and oropharynx: Natural history-cellular and molecular markers of risk. Eur J Cancer Prev.

[2] Mehrotra R, Gupta A, Singh M, Ibrahim R (2006). Application of cytology and molecular biology in diagnosing premalignant or malignant oral lesions. Mol Cancer.

[3] Bindu L, Balaram P, Mathew A, Remani P, Bhattathiri VN, Nair MK (2003). Radiation-induced changes in oral carcinoma cells: A multiparametric evaluation. Cytopathology.

[4] Bindu L, Balaram P, Mathew A, Remani P, Bhattathiri VN, Nair MK (2003). Radiation-induced changes in oral carcinoma cells: A multiparametric evaluation. Cytopathology.

[5] Mehrotra R, Goel N, Singh M, Kumar D (2004b). Radiation-related cytological changes in oral malignant cells. Indian J Pathol Microbiol.

[6] Scully C, Sonis S, Diz PD (2006). Effect of granulocyte-macrophage colony-stimulating factor on chemotherapy-induced oral mucositis in non-neutropenic cancer patients. Oral Dis.

[7] Sonis ST (1998). Mucositis as a biological process: A new hypothesis for the development of chemotherapy-induced stomatotoxicity. Oral Oncol.

[8] Maraki D, Boecking A, Pomjanski N, Megahed M, Becker J (2006). Cytologic and DNA-cytometric examination of oral lesions in lichen planus. J Oral Pathol Med.

[9] Ogden GR, Cowpe JG, Chisholm DM (1991). Cost of oral screening. Lancet.

[10] Ahmed HG, Idris AM, Ibrahim SO (2003). Study of oral epithelial atypia among Sudanese tobacco users by exfoliative cytology. Anticancer Res.

[11] Ogden GR, Cowpe JG, Wight AJ (1997). Oral exfoliative cytology: Review of methods of assessment. J Oral Pathol Med.

[12] Umiker W, Lampe I, Rapp R (1959). Irradiation effects on malignant cells in smears from oral cancers: A preliminary report. Cancer.

[13] Tucker JH, Cowpe JG, Ogden GR (1994). Nuclear DNA content and morphometric characteristics of normal, premalignant and malignant oral smears. Anal Cell Pathol.

[14] Ramaesh T, Ratnatunga N, Mendis BR, Rajapaksa S (1998). Exfoliative cytology in screening for malignant and premalignant lesions in the buccal mucosa. Ceylon Med J.

[15] Cowpe JG, Longmore RB, Green MW (1988). Quantitative exfoliative cytology of abnormal oral mucosal smears. J R Soc Med.

[16] Vaillant JM (1994). Screening and diagnosis of epidermoid carcinoma of the oral mucosa: What conclusions are possible today concerning the respective roles of cytologic smear and biopsy?. Bull Acad Natl Med.

[17] Kaugars GE, Silverman S, Ray AK, Page DG, Abbey LM, Burns JC (1998). The use of exfoliative cytology for the early diagnosis of oral cancers: Is there a role for it in education and private practice?. J Cancer Educ.

[18] Man YG, Nieburgs HE (2006). A subset of cell clusters with malignant features in morphologically normal-appearing and hyperplastic tissues. Cancer Detect Prev.

[19] Ogden GR, McQueen S, Chisholm DM, Lane EB (1993). Keratin profiles of normal and malignant oral mucosa using exfoliative cytology. J Clin Pathol.

[20] Kumari R, Chaugule A, Goyal PK (2005). Karyoanomalic frequency during radiation therapy. J Cancer Res Ther.

[21] Sonis ST, Fey EG (2002). Oral complications of cancer therapy. Oncology (Williston Park).

[22] Montgomery MT, Redding SW, LeMaistre CF (1986). The incidence of oral herpes simplex virus infection in patients undergoing cancer chemotherapy. Oral Surg Oral Med Oral Pathol.

[23] Devine SM, Wingard JR (1994). Viral infections in severely immunocompromised cancer patients. Support Care Cancer.

[24] Wilkes JD (1998). Prevention and treatment of oral mucositis following cancer chemotherapy. Semin Oncol.

